# Comparison of plaque control record measurements obtained using intraoral scanner and direct visualization

**DOI:** 10.1002/cre2.852

**Published:** 2024-02-01

**Authors:** Kazuya Doi, Chihiro Yoshiga, Hiroshi Oue, Reiko Kobatake, Maiko Kawagoe, Hanako Umehara, Kaien Wakamatsu, Kazuhiro Tsuga

**Affiliations:** ^1^ Department of Advanced Prosthodontics Hiroshima University Graduate School of Biomedical and Health Sciences Hiroshima Japan; ^2^ Department of Dental Hygiene Hiroshima University Hospital Hiroshima Japan

**Keywords:** intraoral scanner, plaque control record, plaque disclosing

## Abstract

**Objective:**

Intraoral scanner (IOS) can acquire three‐dimensional color images of teeth. Thus, the detection of areas with plaque adhesion stained by plaque‐disclosing solutions using an IOS could be a potential oral hygiene evaluation method. This study aimed to verify the usefulness of obtaining O'Leary's plaque control record (PCR) measurements using an IOS in clinical practice.

**Methods:**

Twenty patients with >20% PCR measurements who underwent oral prophylaxis were enrolled in this study. A plaque‐disclosing gel was applied to stain the areas with plaque adhesion, and the dentition was scanned using the IOS. The PCR values obtained via the direct method and those obtained using the digital image were compared for the entire dentition, maxillary total area, the labial and palatal aspects of the maxillary anterior teeth, the buccal and palatal aspects of the maxillary posterior teeth, mandibular total area, the labial and lingual aspects of the mandibular anterior teeth, and the buccal and lingual aspects of the mandibular posterior teeth.

**Results:**

The IOS group tended to have higher values than the direct observation group. The labial and palatal aspect of the maxillary anterior teeth, the labial and lingual aspects of the mandibular anterior teeth did not differ significantly between the groups.

**Conclusion:**

Plaque adhesion was visualized easily and conclusively using an IOS. As the large tip size often hinders its use, it is necessary to develop a smaller IOS tip in the future.

## 1 INTRODUCTION

Biofilm formation due to the adhesion of dental plaque on the tooth surfaces is a risk factor for the incidence of periodontal disease and dental caries (Costalonga & Herzberg, 2014; Larsen & Fiehn, 2017; Tonetti et al., 2015). Plaque removal plays a crucial role in the prevention of periodontal disease and dental caries. Thus, plaque control through daily self‐care should be performed to prevent periodontal disease (Figuero et al., 2017; Needleman et al., 2015; Tonetti et al., 2015). Tooth brushing can effectively remove dental plaque. Therefore, it is important to accurately assess the state of plaque adhesion in the oral cavity and provide appropriate oral hygiene instructions to patients to ensure adequate maintenance of oral hygiene through daily tooth brushing.

Plaque‐disclosing solutions can be used to visualize dental plaque. The red or blue dye from the solution binds to the proteins and polysaccharides in the dental plaque, leading to visualization of the plaque (Datta, 2017). Plaque‐disclosure methods are widely used in dental hygiene evaluations as they aid in visualizing intraoral biofilms, which are mostly colorless and transparent (Pretty et al., 2005). O'Leary's plaque control record (PCR) has been used in clinical practice as a method for evaluating plaque control (O'Leary et al., 1972). In PCR, the dental plaque in the oral cavity is stained using plaque‐disclosing solutions, and the presence of the plaque is confirmed visually. The tooth surface is then divided into several blocks, and the ratio of the plaque‐adherent areas is calculated.

This method has the advantages of simplicity and only requiring visual evaluation; however, it is also associated with disadvantages, such as interoperator variability, that is, differences in the findings of evaluations performed by different surgeons, and the inability to record findings. Moreover, the lingual surface and molars' distal surface are difficult to visualize and evaluate with mirrors; therefore, they are often overlooked. The use and evaluation of intraoral digital images may remedy this disadvantage.

Remarkable progress has been made in the field of digital dentistry in recent years. Intraoral scanner (IOS) can record digital images of the dentition in the mouth, and they have been used in the fabrication of crowns and prostheses using the computer‐aided design/computer‐aided manufacture technique (Fine, 1995). The digital images recorded by IOS can be rotated and magnified on the monitor, and dentition can be observed from angles that are not normally visible (Patzelt et al., 2014). Thus, taking advantage of this feature, attempts have been made to use IOS to evaluate oral hygiene conditions.

Our previous study was the first to report that color‐displaying IOS can detect stained plaque adhesions (Doi et al., 2021). PCR of the stained dental plaque was measured on the monitor using an IOS, and the PCR value was found to be higher than that of the conventional visual method. Thus, the detection of plaque‐encrusted areas using an IOS may be useful as a new oral hygiene evaluation method. Reports on oral hygiene conditions evaluated using IOS have increased in number, indicating that the application of IOS for oral hygiene evaluation is expected to continue increasing (Doi et al., 2021; Giese‐Kraft et al., 2022; Jung et al., 2022). However, the usefulness of IOS‐based oral hygiene assessment in clinical practice is still insufficient. Therefore, this study aimed to verify the usefulness of obtaining PCR measurements using IOS in clinical practice.

## 2 METHODS

### 2.1 Study design and participants

This was an interventional, single‐arm, open (masking was not used), uncontrolled, single‐assignment study. This clinical study was conducted at the Department of Dental Surgery, Hiroshima University Hospital, Japan, from January 2023 to June 2023.

This study was approved by the Hiroshima University Hospital Certified Review Board (CRB6180006). This study was conducted as a clinical research study (jRCTs062220068) in accordance with the Good Clinical Practice Guideline of the International Conference on Harmonization (ICH‐GCP).

The study included 20 patients who underwent oral prophylaxis (7 males and 13 females; age, 25–85 years). All patients received information regarding the study protocol and provided informed consent for undergoing treatment procedures along with PCR measurements using an IOS.

The inclusion criteria were as follows: age ≥18 years, written informed consent provided, presence of periodontal disease or peri‐implantitis, underwent regular oral treatment performed by dentists or hygienists (>6 months), PCR > 20%, and cognitive ability to understand and answer questionnaires accurately.

The exclusion criteria were as follows: individuals with pacemakers or ICDs, presence of edentulous arches, absence of all four molars and premolars, mouth‐opening or temporomandibular disorders, and　those deemed inappropriate for inclusion by the principal investigator or coinvestigators.

### 2.2 Clinical procedure

Patients were seen under usual self‐hygiene conditions. Plaque staining was performed using a plaque‐disclosing gel (PROSPEC; GC Corporation). The dental plaque staining status in the oral cavity was recorded using an IOS (TRIOS 3^Ⓡ^ Basic; 3Shape) as color images.

### 2.3 Clinical procedures

#### 2.3.1 Plaque staining and recoding

The extent of plaque adhesion was assessed by staining the dental plaque with the staining solution and visualizing it with an IOS to evaluate the oral hygiene status. A cotton ball soaked in the plaque‐disclosing gel was used to apply the solution to the tooth surface. The patients then rinsed their mouths with water. The dentition was then recorded using the IOS after staining. Then, the buccal sides of the artificial teeth were recorded using a digital single lens reflex camera (Camera; EOS 60D; Canon).

#### 2.3.2 Evaluation of plaque adhesion state

After recording the staining status, the PCR values were measured via direct observation or using a dental mirror in the dentist's chair (direct method). Later, the PCR values were measured on an IOS digital image in the monitor. These PCR measurements were performed by O'Leary's PCR method. The PCR values were evaluated by calculating the percentage of the total area of the tooth surfaces with plaque adhesion on the cervical area. PCR measures the axial surface of the tooth in six sections; mesial, central, and distal surfaces at the buccal sides, and on the mesial, central, and distal surfaces at the lingual side.

#### 2.3.3 Comparison of PCR

The PCR values were obtained using the direct method and IOS digital imaging. The PCR values were compared for the entire dentition, the maxillary total area, the labial and palatal aspects of the maxillary anterior teeth, the buccal and palatal aspects of the maxillary posterior teeth, the mandibular total area, the labial and lingual aspects of the mandibular anterior teeth, and the buccal and lingual aspects of the mandibular posterior teeth. Differences between the PCR values of direct method and IOS method were analyzed using the Wilcoxon matched‐pair signed‐rank test. The calculations were performed using Prism v7 (GraphPad), and a *p*‐value < .01 were considered statistically significant.

#### 2.3.4 Ethics statement

This clinical study was approved by the Hiroshima University Hospital Certified Review Board (CRB6180006). This study was conducted as a clinical research study (jRCTs062220068) in accordance with the Good Clinical Practice Guideline of the International Conference on Harmonization (ICH‐GCP). All patients received information regarding the study protocol and provided informed consent for undergoing treatment procedures.

## 3 RESULTS

### 3.1 Plaque observation

**Figure 1:**
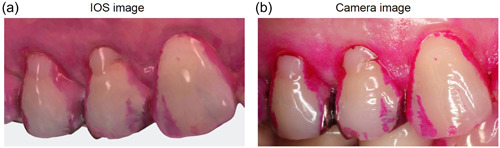
IOS and camera images after dental plaque stained. (a) IOS image, (b) camera image. IOS, intraoral scanner.

**Figure 2:**
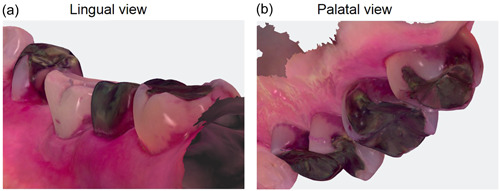
IOS images from various angles. (a) Lingual view, (b) palatal view. IOS, intraoral scanner.

The stained plaque was visualized similarly in the images acquired using the IOS (Figure 1a) and the camera (Figure 1b). In the IOS image, the color appeared slightly pale red (Figure 1a). The IOS images can be used to directly observe the lingual (Figure 2a) and palatal sides (Figure 2b).

### 3.2 Comparison of PCR

The PCR results are shown in Table 1. The PCR values obtained using the IOS were significantly higher than those obtained using the direct method.

**Table 1:** Subjects information and measurements data.

Subjects	Age	Sex	PCR (%)		Excluded teeth
			Direct	IOS	FDI two‐digit system
1	85	F	24	29	
2	67	F	61.4	70.5	38
3	71	M	82	95	
4	25	M	53	62.9	
5	75	M	36	53.3	18, 28, 38, 47
6	47	M	29.7	42	17, 27, 37
7	75	F	59.1	66.7	
8	66	F	21	40.7	
9	75	M	72.8	86	
10	83	M	40.3	55.6	37
11	74	F	39.2	64.7	
12	54	F	70	82.6	
13	77	F	60	83	
14	66	F	25	42.5	37
15	58	F	67.3	71.6	37
16	74	F	45.7	67.4	
17	80	F	67.9	77.8	
18	74	M	34.5	42.9	
19	61	F	30	41.2	
20	61	F	36.5	47.6	18, 38, 48

*Note*: PCR values of direct method and IOS method.

Abbreviations: IOS, intraoral scanner; PCR, plaque control record.

### 3.3 Site comparison of PCR

The PCR values were compared according to the site. Table 2 presents the PCR values obtained using the direct method with those obtained using the IOS. A comparison of the PCR values at the maxillary site revealed no significant differences between the labial or palatal aspects of the anterior teeth. In contrast, the PCR values obtained using the IOS were significantly higher than those obtained using the direct method for the buccal and palatal aspects of the posterior teeth. No significant differences were observed between the labial or lingual aspects of the mandibular anterior teeth. In contrast, the PCR values obtained using the IOS were significantly higher than those obtained using the direct method for the buccal and lingual aspects of the posterior teeth.

**Table 2:** Comparison of PCR value by site.

Maxillary				Mandibular			
Anterior teeth		Posterior teeth		Anterior teeth		Posterior teeth	
Labial	Palatal	Buccal	Palatal	Labial	Lingual	Buccal	Lingual
No significance	No significance	IOS*>direct	IOS*>direct	No significance	No significance	IOS*>direct	IOS*>direct

Abbreviations: IOS, intraoral scanner; PCR, plaque control record.

*p < .001.

The site‐specific comparison revealed that the PCR values obtained using the IOS were significantly higher in the posterior region in the maxilla and mandible; however, no such difference was observed in the anterior teeth with the use of the direct method.

### 3.4 Excluded measurement sites

PCR values of some areas could not be obtained due to the inability of the IOS to capture images. These areas were mainly located in the posterior molars (Table 1).

The appearance of the stained plaque was very similar in both images. In the IOS image, the color appeared slightly pale red.

The IOS images can be used to directly observe from the lingual and palatal sides.

## 4 DISCUSSION

Brushing instructions play a crucial role in achieving good plaque control (Husseini et al., 2008). The oral cavity should be stained with a plaque‐staining solution to visualize the areas with plaque deposition, and demonstrations should be given using a jaw model to ensure that patients follow proper brushing instructions. The evaluation methods used in clinical practice for assessing plaque control should possess the following characteristics: highly objective evaluation; quantification; simple and accurate examination and recording; high reproducibility; and easy understanding of the evaluation by the operator and patient.

PCR, which was published by O'Leary et al. in 1972, is a method in which the practitioner evaluates the stained area of dental plaque in the oral cavity by direct observation or using a dental mirror. As an oral hygiene evaluation method, it is widely used in clinical practice and research to compare the effects of treatment (O'Leary et al., 1972). However, this method has been concerned that the PCR method shows differences in the measurement values owing to differences in evaluation criteria among practitioners, even in the same patient with each visit. Moreover, it is not possible to record plaque adhesion findings at the time of evaluation.

The solution to these problems is the acquisition of a simple digital image of the dentition.

Recording the oral cavity with an optical camera can indirectly aid in the evaluation of the oral hygiene status; however, considering the time required for filming, the need for assistants, and the burden on the patient, this method is burdensome for both the practitioner and patient at every maintenance visit.

An IOS can record intraoral conditions as digital images, and measurements can be performed by a single operator. Moreover, the burden on the patient is low. Intraoral assessment using IOS has been reported in recent years (Giese‐Kraft et al., 2022; Jung et al., 2022) IOS, which can be color‐coded, is used to assess the plaque adhesion status.

Giese‐Kraft et al. (2022) compared images recorded using an intraoral camera and IOS after plaque staining on a monitor and reported that the results were very similar.

Jung et al. (2022) reported that the entire tooth surface could not be observed in the central image owing to the convexity of the tooth surface in the image acquired by the intraoral camera; however, the entire tooth surface could be visualized on the IOS image. Therefore, the amount of plaque visualized on the IOS image was slightly greater than that visualized on the intraoral camera image.

In our previous study that examined the utility of IOS for PCR measurements, the IOS image obtained using IOS clearly revealed the presence of stained dental plaque (Doi et al., 2021). Rotating the image aided in visualizing the stained plaque, especially in areas that were difficult to observe directly, such as the lingual and most centrifugal posterior molar areas.

The results showed that the PCR values obtained using IOS were higher than those obtained using direct evaluation. The IOS evaluation of the posterior teeth yielded higher values than those obtained using direct evaluation. Areas with significant differences were difficult to view directly and had to be visualized using a dental mirror. Therefore, the field of view to be evaluated was considered to have become narrower, and the state of plaque adhesion could not be detected. The IOS image enabled magnification and rotation, which facilitated measurement even in areas that were difficult to view directly.

This aspect is corroborated by a subsequent study that reported that intraoral digital monitors are considered more useful than dental mirrors for assessing stained plaque and tartar in areas that are difficult to visualize directly (Levi et al., 2016). And Meseli et al. (2023) reported that Intraoral digital scanning offers a distinct advantage in diagnosing dental plaque due to its three‐dimensional imaging capabilities.

In contrast, no difference was observed in the PCR measurements of the anterior teeth obtained using IOS and direct viewing. This may be attributed to the fact that these areas could be viewed directly, and there were no problems with obtaining PCR measurements.

These results suggest that the IOS is useful for assessing the oral hygiene status. IOS can aid in accurately evaluating the status of dental plaque adhesion by visualizing it on the monitor, especially in the observation of areas that cannot be viewed directly and require dental mirrors. In addition, images can be acquired easily, enabling transitional evaluation and evaluation by multiple evaluators, which is more effective than conventional techniques.

However, oral hygiene assessment using IOS has some limitations. Some teeth could not be scanned using the IOS in the present study. This was especially true for most posterior molars. The large tip size of the current IOS may make it difficult to place it in the imaging position when the mouth opening is limited. The placement is also affected by the height of the masticatory muscles and the crest of the jaw. Therefore, it is necessary to develop a smaller tip for recording the entire dentition.

## 5 CONCLUSION

The present study shows that PCR measurements for oral hygiene evaluation could be performed more accurately using the IOS; however, measurements could not be acquired in some cases, suggesting the need to conduct further studies.

## AUTHOR CONTRIBUTIONS

Chihiro Yoshiga and Kazuya Doi contributed to the study concept and design. Maiko Kawagoe and Hiroshi Oue participated in the data collection and Hanako Umehara, Kazuya Doi, and Kaien Wakamatsu checked the data. Reiko Kobatake performed the statistical analyses. Chihiro Yoshiga, Kazuya Doi, and Reiko Kobatake drafted the manuscript. Chihiro Yoshiga, Kazuya Doi, Hiroshi Oue, Maiko Kawagoe, Reiko Kobatake, Hanako Umehara, Kaien Wakamatsu, and Kazuhiro Tsuga reviewed the findings of data analyses, participated in writing the manuscript, and approved the final draft of the manuscript submitted to the journal.

## CONFLICT OF INTEREST STATEMENT

The authors declare no conflict of interest.

## Data Availability

All data generated or analyzed during this study are included in this published article.
